# A mathematical model for thrombotic risk assessment in type 2 diabetes

**DOI:** 10.6026/97320630019971

**Published:** 2023-09-30

**Authors:** Sahana Kashyap, Indumathi AN, Shashidhar KN, Harish R

**Affiliations:** 1 Department of Biochemistry, Sri Devaraj Urs Medical College, Constituent college of Sri Devaraj Urs Academy of Higher Education and Research, Tamaka, Kolar, Karnataka, India

**Keywords:** Diabetes mellitus, Castelli's risk indices, dyslipidemia

## Abstract

Hyperglycaemia is known to alter the circulating lipids in diabetics. Combinatorial effect of *in vivo* synthesis of lipids and dietary lipids leads to atherosclerosis. Uncontrolled diabetes is linked with the cardiovascular outcome. This data has correlated the Castelli's Risk Index (CRI-I and CRI-II), Atherogenic Index of Plasma and Atherogenic Coefficient with microvascular complications of T2DM. Etio-pathogenesis of cardiovascular risk factors and lipid biomarkers speaks of the thrombotic events of cerebrovascular accidents and also the reno-vascular mechanisms of renal arterial thrombotic events. Documentary evidence have proved that the micro albuminuria is a "cutting edge" to assess the microvascular complications of renal and retina. Uncontrolled diabetes is known to alter the triglycerides, lower HDL-cholesterol and elevate LDL-cholesterol. Alteration of lipid profile mimics a major link between diabetes and the increased cardiovascular risk in diabetic patients.

## Background:

Diabetes mellitus is a complex metabolic disorder characterized by Hyperglycaemia [1]. Hyperglycaemia results from alterations in either insulin synthesis or insulin action or receptor. [2]. Triglyceride rich lipoproteins metabolism results in alteration of insulin mediated pathways of free fatty acids, HDL, LDL and squeal of inflammatory changes in the arteries [3]. These changes are overcome by rigid lifestyle modification and glycaemic control. Stains prevent cardiovascular risk in high-risk category individuals [4]. Around 65% of cardiovascular deaths in diabetes are due to the coronary microvascular complications. Dyslipidemia is a front runner in development of atherosclerosis. Derangement in lipid profile and atherogenic indices are early predictors of the pathogenic mechanisms of diabetes. Early detection of the deranged lipids and atherogenic indices shall devise the treatment modalities to help prevent development of CVD in diabetes [5]. Scientific evidence predicts a strong association of elevated LDL with low HDL in CVD. Increased LDL-C/HDL-C ratio indicates cardiovascular risk [6]. Lipid ratios viz Castelli Risk Index - I, II has a better prognostic value in the prediction of cardiovascular risk compared to HDL and/or LDL levels. AIP is also proposed to be yet another predictor of atherogenicity [7]. Therefore, it is of interest to calculate the lipid ratios using the mathematical models of Castelli Risk Index and AIP in patients with type 2 diabetes mellitus.

## Materials and methods:

Under aseptic precautions, 3ml of venous blood was collected from the median orbital vein in sitting position. Fasting blood was collected for FBS and lipid profile, 2 hours post prandial blood was collected for PPBS. Written informed consent was taken from all study subjects. Ethical clearance was taken from the study subjects before the start of the study. One hundred and forty subjects in the age group of 30-70 years were included in the study. It was confirmed that the study subjects were free of other comorbidities. Blood glucose was estimated by glucose oxidase and peroxidase, HDL by precipitation method, TG by glycerol kinase method, TC by cholesterol oxidase-peroxidase method. All the investigations are carried out by Vitros 5.1 FS dry chemistry analyser based on principle of reflectance photometry.

## Castelli's Risk Index (CIR):

Castelli's Risk Index (CRI) was calculated with TC, LDLc and HDLc and categorized into two groups; CRI -I and CRI -II. CRI-I includes TC/HDLc. CRI-II by LDLc/HDLc.

## Statistical analysis:

## Atherogenic Index of Plasma (AIP) by Log10 (TG/HDLc):

Atherogenic Coefficient (AC) by [(TC- HDLc)/HDLc] or [(Non-HDLc)/HDLc]. Statistical analysis was done using licensed version of SPSS. Descriptive statistics was considered for calculating the mean ± SD.

## The test result variable(s):

TESTAIP has at least one tie between the positive actual state group and the negative actual state group. Statistics may be biased. a. Under the nonparametric assumption b. Null hypothesis: true area = 0.5.

## Results:

**Figure 1:**
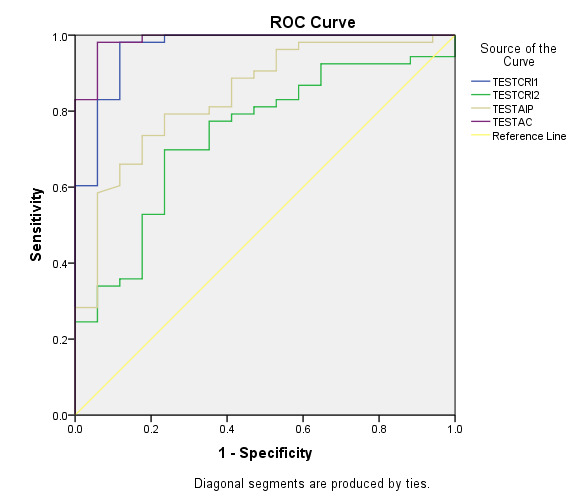
Receiver operating characteristics curve analysis of lipid indices in patients with diabetes mellitus type 2 for thrombotic risk assessment with controls. ROC- receiver operating curve, CRI-I: Castelli's risk index I, CRI-II: Castelli's risk Index II.

Age and gender matched study subjects with mean of 49.22±1.11 for controls and 51.34±1.01 for DM patients with p-value 0.211 were documented. Lipid ratios of patients with diabetes mellitus (DM) were compared with healthy controls (Table 1). Present study was designed to assess the benefits of lipid ratios derived from basic lipid profile in diabetic patients in the risk assessment of cardio metabolic conditions. Total cholesterol (p= 0.001), triglycerides (p= 0.001) and low-density cholesterol (p= 0.003) were significantly higher in cases compared to the controls. HDL values were decreased in diabetes compared to control group (p=0.891); FBS, PPBS and HbA1c were found to be significantly higher in cases compared to the control group with p-value of 0.001 for all the three groups. CRI-I, CRI-II, AC and AIP were found to be increased in cases compared to controls with p-value 0.001for CRI-I and AIP. The p value was 0.003 and 0.011 for CRI-II and AC respectively. Pearson correlation of lipid parameters with lipid indices was applied and values are depicted in table 2. Ratios CRI-I, CRI-II, and AIP correlated positively with Total cholesterol with r and p value of 0.512, 0.001; 0.392, 0.001 and 0.176, 0.001. AC showed negative correlation with total cholesterol with r= -0.138, p= 0.001. Lipid indices showed significant negative correlation with HDL cholesterol with r= -0.678, p <0.001; r= -0.413, p <0.001; r= -0.326, p <0.001; r= -0.455, p <0.001. AIP and AC was documented to be negatively correlated with LDL cholesterol with r= -0.467, p= 0.001 and r= -0.154, p= 0.001 respectively. CRI-I and CRI-II showed positive correlation with LDL of r= 0.764, p= 0.001 and r= 0.060, p= 0.001 respectively. CRI-II showed significant negative correlation with TG of r= -0.031, p= 0.001 and CRI-1, AIP, AC indices showed positive correlation. CRI-1 and AIP showed positive association with FBS, PPBS and HbA1c (Table 3). CRI-II and AC showed negative correlation with FBS, PPBS and HbA1c. Table 4 denotes the correlation of lipid indices and diabetic profile in T2DM compared with non-diabetics. CRI-I, CRI-II, AC and AIP were observed to have significant diagnostic ability to detect the presence of thrombotic risk as determined by using ROC. The AUC (figure 1) for AC and CRI-I were higher and stastically significant compared to other lipid parameters of 0.988, 0.964 respectively.

## Discussion:

Diabetes mellitus plays a significant role in lipid metabolism. Current study observed that there is a proportionate correlation of TG in both cases and controls. However, there is a marginal elevation of TG in patients with DM of 1.13:1. This good correlation may be attributed to the dietary regulation and lifestyle modification. TG was estimated in fasting condition. We correlated TG with PPBS values to find if any contribution of the glucose via triacylglycerol and phospholipid biosynthesis pathway. Further, we also tried to find if glycerol-3-phosphate and dihydroxyacetone phosphate derived from glycolysis were been diverted to the TG by in-vivo synthesis. We observed a negative corelation in type 2 DM compared to controls, indicating the TG values is independent of the post prandial diabetic status. Our studies partially correlate with the studies conducted by Banday.et.al. [1].

PPBS vs TC in cases and control are 39 and -17. This positive values in cases and negative values in controls are contributed to the totality of the Acetyl CoA derived from the glycolysis, sequel of hyperglycaemia, ketone body derived, and beta oxidation of lipids or other sources. Since these patients were not on statins or hypolipidemic drugs, comment on HMG CoA reductase or HMG CoA synthase regulation by the glycemic hormones cannot be commented. FBS vs HDL in cases and controls, we could not find significant differences and HDL was observed to be within the biological reference interval. However, to our surprise PPBS vs HDL in cases vs controls is 2.7:1 indicating that any increase in PPBS there is a proportionate elevation in HDLc contributing to the protecting benefits of HDL. We compared LDL values in both fasting and post prandial condition in cases and controls. There is a significant increase in LDL in cases compared to controls. Our observation with respect to LDL: PPBS in cases vs controls is 2:1. This indicates a higher transportation of LDL to the peripheral tissues but with a proportionate reflex response by the HDL in reverse cholesterol transport. Further LDL:HDL ratio in cases and controls is around 0.6 and is statistically significant with p value of 0.0001.

TG:HDL ratios in cases vs control, it is 2:1 indicating doubled TG values in cases correlating well with the blood glucose value. These elevated values can be correlated with the in-vivo synthesis of TG from glucose in addition to dietary supply. However, we observed the LDL: PPBS and TG: HDL are 2:1. TG: TC ratio is observed to be 1.3:1 time indicating that in addition to the acetyl CoA derived from pyruvate the end product of glycolysis, there is a marginal contribution of acetyl CoA from other sources [8]. Non-HDL:HDL ratio we observed 4:1 in cases vs control. This indicates indirectly that the TC values are four times higher in cases vs controls contributing a higher chance of complications related to hypercholesteremia in diabetics compared to controls. CRI-I (TC: HDL) and CRI-II (LDL: HDL), we did not observe any difference and the values were same and it was 1.2:1. This indicates that there is no much difference between CRI-I and CRI-II and both have equal importance with respect to diabetics. AIP a calculated parameter we got a value of 1.6:1. Calculated AC values we derived cases vs controls 2.4:1, indicating 2.4 times higher non-HDL compared to HDL. Lipid indices CRI-I, CRI-II, AC and AIP were found significantly correlated with lipid parameters. Lipid indices demonstrated a positive correlation with Total cholesterol and negative correlation with HDL. AIP positively correlated with triglycerides. FBS, PPBS and HbA1c are shown to have positive correlation with CRI-I and AIP, indicating relevance of the risk predictors over individual lipid parameters [9].

AIP show highest positive correlation with TG and diabetic parameters. Studies have documented major adverse cardiovascular events. AIP was independently and positively correlated with a high risk among non-diabetic hypertensive adults [10, 11]. Our studies are consistent with the studies conducted elsewhere, indicating index ratio can be an accepted sensitive biomarker of atherosclerotic CVD risk as it reveals the presence of atherogenic small LDL particles. The LDL/HDL or CRI-II predicts risk of heart disease which is better than the unitary evaluation of LDL. Studies have confirmed that CRI-II is a meritorious measure to assess the effectiveness of lipid lowering therapies and denotes greater predictive marker compared to only lipid parameter estimation in cardiovascular diseases as outcome [12]. Present study predicts a significant positive association between the lipid ratios. Vis-à-vis TC/HDL or LDL/HDL. Thus, CRI-I and CRI-II predicts future cardiovascular events in diabetics. [13].

## Conclusion:

CRI-I and CRI-II, AC and AIP are better predictors for assessment of cardio metabolic risk in diabetics compared to traditional lipid estimations. However, small sample size limits to derive any strong conclusion.

## Figures and Tables

**Table 1:** Comparison of biochemical parameters in clinically proven healthy controls and T2DM

**Parameter**	**Controls (n=70) Mean±SE**	**Patients with DM (n=70) Mean±SE**	**Biological Reference range**	**P-value**
TG (mg/dL)	123.07±6.44	201.35±12.12	44- 150	0.001*
TC (mg/dL)	148.51±5.98	193.27±5.08	120-200	0.001*
HDL (mg/dL)	42.74±1.26	42.42±1.89	40-60	0.891
LDL (mg/dL)	95.07±4.58	117.95±5.88	100-169	0.003*
FBS (mg/dL)	91.57±1.79	168.48±9.87	70-100	0.001*
PPBS (mg/dL)	113.41±2.11	231.54±9.87	80-130	0.001*
HbA1c	5.34±0.53	7.65±0.18	9-Apr	0.001*
Non-HDL (mg/dL)	105.77±6.04	249.51±5.54	<130	0.001*
CRI-I	3.65±0.17	4.93±0.19	≥5.0	0.001*
CRI-II	2.23±0.12	2.95±0.16	≥3.0	0.003*
AIP	3.15±0.22	5.34±0.42	≥0.24	0.001*
AC	63.02±6.36	4.65±0.75	≥0.24	0.011*
FBS; Fasting blood sugar, PPBS; Post prandial blood sugar, TG; Tri glycerides, TC; Total cholesterol, LDL; Low density lipoprotein Cholesterol, HDL; High density Lipoprotein cholesterol, GOD; Glucose Oxidase, POD; Peroxidase, CRI; Castelli's Risk Index, AIP; Atherogenic Index of Plasma, AC; Atherogenic Coefficient. Data presented as Mean ± Standard error. <0.05 is considered as statistically significant. N: Number of samples, mg/dL: Milli gram per decilitre, µg/dL: Micro gram per decilitre, µIU/mL: Micro international unit per mille litre. Biological reference interval: NCEP, ATP III guidelines and ADA.

**Table 2:** Correlation of lipid indices with lipid parameters

**Parameter**	**TG**		**TC**		**HDL**		**LDL**	
	r-value	p-value	r-value	p-value	r-value	p-value	r-value	p-value
CRI-I	0.403**	0.001*	0.512**	0.001*	-0.678**	0.001*	0.06	0.001*
CRI-II	-0.031	0.001*	0.392**	0.001*	-0.413**	0.001*	0.764**	0.001*
AIP	0.867**	0.001*	0.176	0.001*	-0.455**	0.001*	-0.467**	0.001*
AC	0.036	0.001*	-0.138	0.001*	-0.326	0.001*	-0.154	0.001*
**: statistically significant, *: significant

**Table 3:** Correlation of lipid indices and diabetic profile in T2DM

**Parameter**	**FBS**		**PPBS**		**HbA1c**	
	r-value	p-value	r-value	p-value	r-value	p-value
CRI-I	0.031	0.001*	0.099	0.001*	0.031	0.001*
CRI-II	-0.084	0.001*	-0.08	0.001*	-0.084	0.001*
AIP	0.112	0.001*	0.165	0.001*	0.112	0.001*
AC	-0.09	0.001*	-0.058	0.001*	-0.09	0.001*
*: Significant

**Table 4:** Area under the Curve

Test results Variables (s)	**Area**	**Std. Error^a^**	**Asympotic Sig.^ b^**	**Asymptotic 95% Confidence Interval**	
				**Lower Bound**	**Upper Bound**
TESTCRI1	0.964	0.025	0	0.915	1
TESTCRI2	0.737	0.067	0.003	0.606	0.868
TESTAIP	0.842	0.053	0	0.737	0.947
TESTAC	0.988	0.011	0	0.966	1

## References

[1] Banday MZ (2020). Avicenna J Med..

[2] Kim H-J, Kim K-I (2022). Diabetes Metab J..

[3] Wu L, Parhofer KG. (2014). Metabolism.

[4] Freeman AM (2022). Stat Pearls..

[5] Chakraborty M (2019). Family Med Prim Care..

[6] Niroumand S (2015). Med J Islam Repub Iran..

[7] Sasikala T, Goswami K. (2020). International journal of clinical biochemistry research..

[8] Huang F (2021). Ren Fail..

[9] Salcedo-Cifuentes M (2019). Arch Med.

[10] Adedokun A (2017). International Journal of Clinical Trials & Case Studies..

[11] Huang F (2021). Ren Fail..

[12] Sastrawan IGG (2022). J penyakit dalam Indones..

[13] Yuan Y (2020). Eur J Clin Nutrition..

